# The Role of Visual Factors in Dyslexia

**DOI:** 10.5334/joc.287

**Published:** 2023-06-29

**Authors:** Árni Kristjánsson, Heida Maria Sigurdardottir

**Affiliations:** 1 Icelandic Vision Lab, Department of Psychology, University of Iceland, IS

**Keywords:** Dyslexia, Reading, Developmental Disorders, Visual Deficits, Visual Perception

## Abstract

What are the causes of dyslexia? Decades of research reflect a determined search for a single cause where a common assumption is that dyslexia is a consequence of problems with converting phonological information into lexical codes. But reading is a highly complex activity requiring many well-functioning mechanisms, and several different visual problems have been documented in dyslexic readers. We critically review evidence from various sources for the role of visual factors in dyslexia, from magnocellular dysfunction through accounts based on abnormal eye movements and attentional processing, to recent proposals that problems with high-level vision contribute to dyslexia. We believe that the role of visual problems in dyslexia has been underestimated in the literature, to the detriment of the understanding and treatment of the disorder. We propose that rather than focusing on a single core cause, the role of visual factors in dyslexia fits well with risk and resilience models that assume that several variables interact throughout prenatal and postnatal development to either promote or hinder efficient reading.

## 1. Introduction

When our car stops on the highway there can be many reasons for the malfunction. One passenger nonetheless insists that the battery is dead and declares that anyone who claims otherwise is wrong. They demand that others must first prove that the reason is not battery malfunction before any alternative options can be investigated, such as that the car may be out of gas, that the spark plugs are faulty, that the petrol car was filled with diesel oil, or whether a combination of these factors contribute to the breakdown. While this scenario is nonsensical, it resembles the field of research on developmental dyslexia surprisingly well.

For most of us, reading is effortless, providing information and entertainment. For readers with dyslexia, it is a struggle. Dyslexia has been defined as a developmental reading disorder that cannot be traced to deficits in general intelligence, neurological deficits, uncorrected refraction problems of the eye, hearing problems, or emotional problems (Shaywitz, 1998). Dyslexia occurs across languages (Richlan, 2020) and is highly heritable (Doust et al., 2022; Schumacher et al., 2007; Úlfarsson et al., 2017; see Erbeli, Rice & Paracchini, 2022 for a recent review). Dyslexia can have major consequences for life success, negatively affecting educational achievement and lifetime earnings (*Mental Capital and Wellbeing*, 2008). The prevalence of dyslexia has been estimated from 5% to 17.5% (Shaywitz et al., 1994; Shaywitz, 1998).

Many researchers have searched for *the one cause* of developmental dyslexia and have been highly critical of other viewpoints. Single source accounts can be contrasted with multifactorial accounts (e.g., Catts & Petscher, 2022; Grainger et al., 2016; Haft et al., 2016; McGrath et al., 2020; Ozernov-Palchik et al., 2016; Pennington, 2006; Pennington et al., 2012; Peters et al., 2019; van Bergen et al., 2014; Vandermosten et al., 2016) where reading ability is assumed to require the interlocked function of many different mechanisms throughout prenatal and postnatal development, and no single cause is necessary or sufficient for dyslexia to occur. Such accounts are consistent with several theories and models suggesting that reading involves a variety of mechanisms (e.g., Castles & Coltheart, 1993; Coltheart et al., 2001; Hautala et al., 2022; Perry et al., 2010; Ziegler et al., 2008), and accordingly there may be many ways in which reading can go wrong. For example, Castles & Coltheart (1993) argue for at least two varieties of developmental dyslexia, characterized by weaknesses in a lexical route (involving retrieval of phonological information from a mental lexicon containing representations of real words) and a sublexical route (involving the use of letter-to-sound rules). But as Ziegler et al. (2007) point out, accurate visual processing is necessary for normal reading via either route. We agree with multifactorial accounts of developmental dyslexia and focus particularly on recent evidence for the contribution of visual factors to the disorder.

### 1.1 Overview of argument

The main aim of this paper is to highlight the multifaceted aspects of dyslexia and to evaluate various visual factors as potential contributors. Our viewpoint can be summarized in the following statements:

- Similar behavioral patterns can have many causes. No core deficit can explain dyslexia. Dyslexia may reflect nontypical phonological or visual processing, or dysfunction in higher-level mechanisms that supersede modalities.
- For many with dyslexia, problems of information processing are not confined to reading, and dyslexic readers may involve subgroups with different symptoms with considerable overlap.
- Understanding which processing problems cause which symptoms is key to explaining and treating dyslexia.
- Visual factors related to dyslexia come in many flavors. Arguments against the role of one visual factor have little bearing on the role of another.
- Visual problems in dyslexia can reflect differences in reading experience but this is not in opposition to their causal role as development is an interactive process.
- We argue for a multifactorial view of dyslexia and call for longitudinal studies where the contribution of different factors to dyslexia can be dissociated, as well as for adversial collaborations.

## 2. Can the phonological account explain the data?

The causes of dyslexia have been debated for decades. A prominent account of dyslexia is the phonological deficit hypothesis (Catts, 1989; Goswami, 2015; Peterson & Pennington, 2015; Snowling, 1981; Szenkovits et al., 2016). The proposal is that dyslexia is caused by dysfunctional processing of the sounds of oral language which can manifest in different ways, including problems with finding words, remembering verbal material, repeating pseudowords, and extracting phonemes from speech (Peterson & Pennington, 2015; Share, 2021). As learning to read involves mapping phonemic and phonological representations of speech sounds to units of written language, a phonological processing deficit could disrupt this mapping. Notably, many treatment programs for dyslexia have focused on phonological problems (Torgesen, 2005).

Importantly, our intention is not to downplay the significance of this approach since there is good evidence that problems with phonological processing are associated with reading deficits. In a landmark study, Bradley & Bryant (1983) found a considerable relationship between a child’s skill in categorizing sounds and future reading success. Bradley & Bryant suggested that children’s awareness of rhyme and alliteration influences their reading and spelling success and that experiences with rhyme might affect later reading and writing. Further research has shown that children with developmental dyslexia are impaired on phonological learning (Wimmer, Mayringer & Landerl, 2000), phonemic awareness (Griffiths & Snowling, 2002) and phonemic fluency (Frith, Landerl & Frith, 1994) and are deficient in verbal short-term memory (Griffiths & Snowling, 2002). Dyslexic readers have trouble with reading pseudowords, where the reading relies on phonology and there can be no aid from context (Rack et al., 1992). Schatschneider et al. (2004) reported that children who score poorly on phonological awareness measures, letter-to-sound knowledge, and naming speed of letters were more likely to develop reading problems. Additionally, children who later develop dyslexia have been shown to have subtle problems with phonological sensitivity, expressive and receptive language, and letter knowledge (Torppa et al., 2010) and their ERP responses to speech sounds differ from those of other children (Guttorm et al., 2005). Additional evidence may come from studies showing how recognition of voices connected with avatars depicting speakers from different races was better for participants’ own race, but importantly that this own-language benefit was absent for dyslexic readers (Perrachione, Del Tufo & Gabrieli, 2011). While this study seemed to point towards impoverished representations of the native language of dyslexic readers, Perea et al. (2014) found that for both children and adults this deficit was independent of language, placing doubts upon a native-language deficit.

A thorny issue for the phonological view is that it does not have great surface validity. Normal (i.e., silent) reading cannot easily be defined primarily as a phonological activity since it involves turning visually presented text into meaning rather than phonological representations. To be clear, we do not claim that phonological representations play no role in reading, silent or otherwise (see discussion by Clifton, pp. 161–176 in Frazier & Gibson, 2015). But importantly, phonological deficits are not found for all of those diagnosed with dyslexia, and dyslexic readers can have other problems that do not involve phonological deficits (Castles & Coltheart, 1993; Hanley & Gard, 1995; Pennington et al., 2012; Peterson et al., 2014; Valdois et al., 2004). Giofrè et al. (2019) tested 300 dyslexic children on the Wechsler intelligence scale for children (WISC-IV). Using cluster analysis, they found two clusters of dyslexic children, one whose deficit was more phonological, while both clusters showed visual processing deficits. O’Brien & Yeatman (2021) tested 106 school-aged children on measures of visual motion processing and standardized measures of phonological processing. When they parcelled the variance in performance due to the different deficits, these factors had largely independent contributions. In Menghini et al. (2010), individual differences that were not related to phonological abilities could account for roughly a quarter of the variance in word reading when age, IQ and most importantly, phonological skills were controlled for.

Stein (2018) goes so far as saying that phonological accounts of dyslexia have, in the end, little explanatory power, but instead simply restate the problem: “the phonological theory only proposes that children fail to learn to read because they fail to acquire the skills required for reading; that is, it could be termed a tautology. Splitting word sounds into their constituent phonemes to match with the written symbols that represent them is the very essence of reading, so the phonological theory seems to say little more, using different words, than that these children cannot read.” Share (2021) argues however that this is a simplification, as splitting speech into phonemes is just one aspect of phonological processing. We agree that phonological problems play a role in dyslexia, but they are unlikely to be the single determining factor behind the disorder. One broken component in an interconnected system can prevent its functioning but different malfunctions can lead to the same result. There are many potential reasons for our car breaking down on the highway and this is true for reading, a highly complex activity that recruits several different mechanisms (e.g., Lesgold & Perfetti, 1981).

Overall, we think it is fair to say that although dyslexia is one of the better-understood learning disabilities, its complexity and multifactorial nature continue to pose challenges for researchers and clinicians. The critical challenge is to find ways of uncovering the variability in symptoms and etiology. While we understand the need for simplicity, the nature of dyslexia may unfortunately be too complex.

Multifactorial accounts of dyslexia have recently gained popularity (Catts & Petscher, 2022; Haft et al., 2016; McGrath et al., 2020; Ozernov-Palchik et al., 2016; Pennington, 2006; Pennington et al., 2012; van Bergen et al., 2014; Vandermosten et al., 2016). For example, Catts & Petscher (2022) proposed a model where dyslexia is attributed to cumulative effects of different risk factors that can be countered by various resilience factors. This idea has good surface validity as even the most fundamental human attributes are influenced simultaneously by various components. For example, a person’s height can be affected cumulatively and interactively by several genes, childhood nutrition, and diseases contracted during development. Take any attribute – let alone something as complex as inclination for fluent reading – and you may assume *a priori* that no single factor can account for individual variability. Children can have propensities for reading problems (e.g., a phonological problem, a visual deficit, or some combination of these) while resilience factors can mitigate the risk of dyslexia (e.g., good phonological abilities could compensate for visual problems). Other aspects are then likely to modulate the influences of such factors. For example, the predictive power of phonemic awareness for reading development appears to be modulated by language, being limited to the first grade in more orthographically transparent languages (Pennington et al., 2012). People with dyslexia do not seem to have any single core deficit (Astle & Fletcher-Martin, 2020; Carroll et al., 2016; Catts et al., 2015; O’Brien & Yeatman, 2021) and children who have both visual and phonological deficiencies are, on average, more likely to develop dyslexia than children with only one deficit (Catts, McIlraith, Bridges & Nielsen, 2017; McGrath, Peterson & Pennington, 2020).

## 3. Problems of vision and related functions in dyslexia

The proposal that visual problems contribute to some cases of dyslexia has high surface validity as efficient reading typically relies on a well-functioning sensory system to accurately process and interpret text. In fact, half of children with dyslexia have been reported to complain of visual problems (Wilkins 1995). The idea that visual problems play a role in dyslexia is certainly not new (see Aaron, 1978; Brachacki et al., 1994; Pontius, 1976, 1983), but has had to keep flailing its arms to keep its head above water. Over a century ago, developmental dyslexia was thought to involve a visual memory deficit (Hinshelwood, 1896; Morgan, 1896). Orton (1925, 1937) studied letter and word reversal errors in dyslexic readers and suggested that they reflected a hemispheric dominance failure where mirror images of visual stimuli were not inhibited. Reading instruction might indeed lead to the loss of mirror invariance for visual words (Dehaene et al., 2010) and dyslexic readers fail to automatize mirror discrimination during visual processing (Fernandes & Leite, 2017).

Any role of visual factors in dyslexia was later dismissed on the grounds that visual acuity in dyslexia is intact, so that dyslexic readers see letters and words as clearly as typical readers do. For example, Vellutino (1979) claimed that intact visual acuity “obviously implies adequacy in basic form perception as well as a physiologically intact visual system”. We disagree. First, equating vision with visual acuity is an oversimplification of the function of the human visual system. Second, vision is not just one ability but involves several different processing mechanisms that often operate independently. Research on visual factors in dyslexia has, in fact, revealed problems at many different levels of visual processing and theories of visual problems in dyslexia therefore come in several flavors. We discuss a few of these here.

### 3.1. Magnocellular dysfunction theory

The primate magnocellular retinocortical pathway is thought to support visual sensitivity at low spatial frequencies (slow changes over space) and high temporal frequencies (transient or fast changes over time; Merigan et al., 1991; Merigan & Maunsell, 1993). It is thought to have high sensitivity to contrast, responding to subtle differences in light vs. dark, but weak color selectivity (Masri et al., 2020). It provides considerable – although not exclusive – input to regions of the dorsal visual pathway, one of the two main cortical visual pathways in primates (Milner & Goodale, 2006; Ungerleider, 1982; Ungerleider & Haxby, 1994; we will turn to the ventral visual pathway below), including cortical regions that support motion perception (Merigan et al., 1991; Merigan & Maunsell, 1993) and attention (see section 3.2). The magnocellular dysfunction theory and magnocellular-dorsal theory suggest that deficiencies in this pathway contribute to dyslexia (e.g., Lovegrove, 1996; Lovegrove et al., 1980; Stein, 2014, 2019).

In an influential paper, Livingstone et al. (1991) found diminished visually evoked potentials to rapid low-contrast stimuli in a group of dyslexic readers. They also reported findings from autopsies where there were abnormalities in magnocellular layers of the lateral geniculate nuclei (LGN) in the retinocortical pathway of dyslexic readers. Slaghuis et al. (1993) reported that visual persistence as a function of spatial frequency differs strongly and interactively between those with and without dyslexia: persistence was longer at lower spatial frequencies but shorter at higher spatial frequencies for dyslexic readers than for control participants. They argued that this reflected deficient processing of the transient visual channels – that have lower spatial resolution – as visual persistence affects the processing of rapid/transient stimuli (see discussion in Lovegrove et al., 1986; and supporting evidence in Ben-Yehudah et al., 2001). Others have also found longer visual persistence for children who have difficulties with reading (Slaghuis & Ryan, 1999; Winters et al., 1989). This is consistent with the idea that these readers may have difficulties with processing rapid sequences of stimuli which is partly handled by the magnocellular system. Consistent with this, Pina Rodrigues et al. (2017) reported that children with dyslexia were impaired at speed discrimination but not at discriminating chromatic contrast, a finding that was interpreted as a specific magnocellular deficit, as the former should heavily depend on the magnocellular-dorsal pathway while the latter should not. Notably, many magnocellular functions appear to be fully developed before children typically start to learn to read (Atkinson, 1992). Recently Flint & Pammer (2019) found magnocellular/dorsal deficits (tested with coherent motion and frequency doubling tasks) for dyslexic readers but not for illiterate, semi-literate, and normal reading adults. Importantly, this ruled out that the deficit is a consequence of reading experience and argued for a causal role of deficient visual magno/dorsal processing in dyslexia. A recent training study (Peters et al., 2021) indicated that dyslexic readers who showed the largest improvement in low contrast magnocellular-temporal processing showed significantly greater improvement in reading accuracy.

But despite many interesting findings, the magnocellular theory faces obstacles, such as low surface validity. Visually presented words are static, high-contrast objects primarily involving high spatial frequency. Lesions of the primate magnocellular retinocortical pathway however reduce contrast sensitivity at high temporal and low spatial frequencies (Merigan et al., 1991) – in other words for blurry fast-moving things but not for static sharp-looking things. In non-human primates at least, the magnocellular pathway makes little contribution to visual sensitivity at low temporal frequencies (Merigan & Maunsell, 1990) and magnocellular lesions cause negligible reductions in flicker resolution for high-contrast stimuli (Merigan & Maunsell, 1993). Skottun (2000) concluded that while several studies showed good evidence for contrast sensitivity reductions in dyslexia, many other studies did not, or showed contrast sensitivity loss primarily for high spatial frequency stimuli. This is problematic for the magnocellular theory as lesions of this pathway in non-human primates are restricted to stimuli containing both high temporal and low spatial frequencies.

Complicating things even further, stimuli that are often thought to excite only the magnocellular pathway may not always do so. For example, when eccentricity is constant, neurons in both the magnocellular and parvocellular pathways appear to have essentially identical spatial resolution (Merigan & Maunsell, 1993). Reading may also become better by removing red light via colored filters, potentially supporting the role of the magnocellular pathway in reading as red light is thought to inhibit the pathway (Chase et al., 2003). However, while red light could affect the magnocellular pathway, its effects are not necessarily specific to this system (Skottun, 2004). Whether colored filters actually help dyslexic readers is in any case controversial (Denton & Meindl, 2016; Razuk et al., 2018; Skottun & Skoyles, 2007; Chase et al., 2003). Behavioral manipulations aimed at targeting magnocellular function (e.g., by manipulating spatial frequency or using colored filters) can therefore be hard to interpret in terms of underlying neurological function.

Finally, some results interpreted in favor of the magnocellular theory may also be consistent with other etiologies. Schulte-Körne & Bruder (2010) reviewed research on visually evoked potentials in response to stimuli designed to target the magnocellular pathway. They concluded that responses to low-contrast rapidly moving stimuli differ between dyslexic and typical readers and that dyslexic readers have visual temporal processing deficits. Instead of a magnocellular deficit they therefore proposed a temporal processing deficit (see also e.g., Ben-Yehudah et al., 2001).

We note that diversity in research findings is not wholly unexpected if dyslexia is a multifaceted disorder, as is the leitmotif in this review. What exactly is found can depend on the participant group tested, or task subtleties. For example, Borsting et al. (1996) reported that only a subset of dyslexic readers had reduced sensitivity to low spatial frequencies at 10 Hz which they interpreted as a magnocellular deficit. But even if such a subgroup exists, the specificity of their problems is debatable. Amitay et al. (2002) reported a subset of dyslexic readers with magnocellular problems, but deficits were additionally found in other perceptual tasks unrelated to magnocellular function; if anything, Amitay et al. (2002) found greater evidence for a non-magnocellular deficit in another subgroup in tasks where fine frequency discriminations were required.

#### 3.1.1. Chicken versus egg problems in development

From a developmental perspective there is an inherent chicken-versus-egg problem with regard to visual factors in dyslexia: Magnocellular-dorsal problems could, for example, be explained by lesser experience with reading (e.g., Goswami, 2015). Olulade et al. (2013) found that brain activity in the motion-selective dorsal stream region V5/MT+ in dyslexic children was similar to that of younger *reading-ability* matched children – supporting the role of experience. However, Gori et al. (2016) then found firstly that visual motion perception was deficient for children with dyslexia, compared to *both* age-matched and reading-level-matched controls. They also reported that motion perception in pre-reading participants predicted future reading problems. This, on its own, could support magnocellular and temporal accounts of dyslexia, but a third finding further strengthened this conclusion where targeted magnocellular function training (without any phonological component), led to improved reading skills in both children and adults with dyslexia (Gori et al., 2016; see Flint & Pammer, 2019; Peters et al., 2021 for supporting findings). Stein (2019) concludes that there is overwhelming evidence that the development of magnocellular neurons is impaired in dyslexia, but the debate will undoubtedly go on. Whether magnocellular dysfunction – or any visual problem – actually leads to reading problems will almost certainly depend on whether other risk factors (e.g., phonological deficits, other language impairments, trauma, poverty, poor instruction) are also present during development and whether resilience factors (various cognitive and socio-emotional protective factors such as family and peer support, sensitivity to contextual information, strong vocabulary, high general intelligence, a growth mindset, or good visual memory) are available (Catts & Petscher, 2022; Haft et al., 2016; Ozernov-Palchik et al., 2016).

### 3.2 Oculomotor and attention problems

Oculomotor and attentional abnormalities of people with dyslexia have often been found. Note that such problems are not always treated as distinct from magnocellular deficits (e.g. Stein & Walsh, 1997), as the dorsal visual pathway, which plays a crucial role in attention and eye movements, receives more magnocellular than parvocellular input (Merigan & Maunsell, 1993; Grainger et al., 2016). These mechanisms play a gating role into other visual regions and magnocellular processing deficits may therefore cause problems in attentional processing (Vidyasagar, 2005).

One notable finding is that dyslexic children are reported to have problems with visual fixation (Stein & Fowler, 1981; Zangwill & Blakemore, 1972). According to Stein and Fowler (1981), 63% of children with dyslexia showed unstable eye dominance while only 1% of typical readers had such problems. More recently, Raghuram et al. (2018) reported that dyslexic participants have difficulties in making vergence eye movements and showed abnormal accommodation and ocular training effects. It is therefore not surprising that many dyslexic readers report that occluding one eye can improve reading (Stein et al., 2000). A more controversial proposal is that dyslexia may be related to general movement deficits (see White et al., 2006, for a critical review).

The causal role of oculomotor abnormalities in dyslexia has relatively good face validity. For example, if dyslexic readers have poor binocular coordination or unstable fixation, this may lead to blurred text, eye strain and fatigue, line skipping, and poor reading comprehension. If such abnormalities precede reading difficulties in dyslexia, unusual oculomotor behavior should also be found in non-reading tasks. Consistently, when viewing paintings, dyslexic readers showed large differences in the amplitude of saccadic movements of the two eyes, pointing to issues with binocular coordination (Kapoula et al., 2009). Children with dyslexia showed more unintended saccadic eye movements than age-matched non-dyslexic children when fixating a circle (Tiadi et al., 2016). When asked to sequentially fixate digits from left to right or right to left, dyslexic readers also looked back more often in both directions than controls (Pavlidis, 1981).

While some studies have reported that dyslexic readers show abnormal oculomotor behavior in non-reading tasks, this claim has not gone unchallenged. For example, while eye movements of dyslexic readers during reading can be distinguished from those of typical readers, no differences were found in eye movement patterns during a task involving the processing of strings of pseudowords (Hutzler et al., 2006). Hutzler et al. therefore concluded that while oculomotor abnormalities might be correlated with dyslexia, they may not be causal. Premeti et al. (2022) discuss various eye movement studies reporting no differences between dyslexic readers and controls. They therefore speculate that oculomotor abnormalities in dyslexic readers are not rooted in visual perception but rather at a higher linguistic level. Several studies however report atypical eye movement parameters of dyslexic readers in both reading and non-reading tasks (Premeti et al., 2022). This involves the number and amplitude of saccades towards a visual stimulus, number and amplitude of backward saccades, number and duration of fixations, and binocular coordination. Whether such abnormalities are causal or not, and whether they reflect low-level or higher-level defects is still under debate.

But even if unusual eye movements are not causal in developmental dyslexia, they could nonetheless offer insights into reading strategies and attentional allocation in dyslexia (Bellocchi 2013; Bellocchi et al., 2013). For example, Hawelka et al. (2010) interpret eye movements in dyslexia in the context of the dual-route model of visual word processing (Coltheart et al., 2001). More specifically, they attributed dyslexic readers’ unusually high fixation numbers and strong word length effect to a lack of orthographic whole-word recognition and an overreliance on serial sublexical processing. As eye movements are tightly connected to the allocation of attention (Kristjánsson, 2011; Craighero & Rizzolatti, 2005; but see Smith & Shenk, 2012), high fixation numbers could also reflect reduced visual attention span, necessitating attention shifts to smaller word segments (see Rayner, 1998).

Dyslexia may partly reflect dysfunctional attentional processing (Grainger et al., 2016; Peters et al., 2019; Valdois et al., 2004, 2019) reflecting different components (orienting, detecting or alerting; Posner & Petersen, 1990). A key observation is that attentional deficits are highly comorbid with dyslexia; the prevalence of Attention-Deficit/Hyperactivity Disorder (ADHD) among people with dyslexia is far higher than among the general population (Beitchman & Young, 1997; Willcutt & Pennington, 2000; Germanò et al., 2010), and the comorbidity may lead to more persistent reading deficits (Willcutt et al., 2007). Importantly, this association is stronger for symptoms of inattention than for symptoms of hyperactivity/impulsivity (Willcutt & Pennington, 2000). McAvinue, Vangkilde et al. (2015), showed that children with ADHD had significantly impaired sustained visual attention and visual processing speed (but intact attentional selectivity, perceptual thresholds, and visual short-term memory capacity). Notably, though, Laasonen et al. (2012) reported that dyslexic adults showed poor performance on temporal and spatial attention, while participants with ADHD did not, suggesting that the same attentional components might not be affected in ADHD and dyslexia.

In an influential paper, Hari & Renvall (2001) proposed that dyslexia involves sluggish attentional shifting (see also Facoetti et al., 2010). Hari et al. (1999) measured this with the so-called attentional blink, where detection of a target hinders the processing of following targets (see e.g. Kristjánsson & Nakayama, 2002; Raymond et al., 1992). However, while Hari et al. (1999) reported that dyslexic readers showed prolonged attentional dwell times in a visual attentional blink task with letters, attending to a target letter actually had the most detrimental effect on the detection of a subsequent target letter at similar time points for both typical and dyslexic readers. The attentional blink had also fully dissipated around the same time for both groups (their figure 2). The attentional blink of dyslexic readers might therefore not actually be longer, as claimed, but stronger. This could indicate that fewer attentional resources are available to dyslexic readers after they process a particular visual stimulus.

Even if dyslexia did not involve sluggish attentional shifting, note that decoding skills involve tight control of attentional shifts across text. According to Vidyasagar & Pammer (2010), dorsal stream attentional mechanisms play a crucial role in the serial scanning of letters. Attention serves as a sequential gating mechanism, enabling orderly processing of letters and words, and dysfunction could lead to reading deficits due to letters not being processed in the proper order which could severely affect the processing of graphemes and their encoding into phonemes, causing phonological symptoms. Vidyasagar & Pammer (2010) point to similarities with the SERIOL model of reading, which also postulates a serial allocation of attention across text (Whitney, 2001). Vidyasagar & Pammer (2010) suggest that reading shares attentional mechanisms with so-called serial visual search, where an attentional spotlight is sequentially directed towards potential targets. However, while dyslexic readers do have problems with serial visual search (e.g., Sireteanu et al., 2008; Vidyasagar & Pammer, 1999), we have recently called into question the assumption that this reflects specific challenges in sequential shifts of attention (Sigurdardottir et al., 2021). This of course does not rule visual attention out as a contributing factor in certain cases of dyslexia. Gavril et al.’s (2021) meta-analysis, found a strong association between visual attention and reading development.

The visual attention span of dyslexic readers seems to be lower than that of typical readers (Bosse & Valdois, 2003; Lobier et al., 2012) – in other words, they appear able to simultaneously process fewer visual elements than typical readers, and this can be amodal (Ahissar, 2007; Facoetti et al., 2010; Goswami, 2011; Lallier et al., 2010; Stein & Talcott, 1999; Hari & Renvall, 2001; Harrar et al., 2014). Lobier et al. (2012) found that the deficit was equal for verbal and nonverbal stimuli which argues that it cannot be explained by phonological problems. It is possible that narrowing the distribution of attention could account for such effects as well as the apparent letter-by-letter reading shown by some dyslexic readers (Ginestet et al., 2019).

Valdois et al. (2004) proposed that phonological and visual attention abilities contribute *uniquely* to reading performance and Franceschini et al. (2012) found that kindergarten children who later became dyslexic made more visual search errors and failed to show a benefit from an attentional cue, suggesting a *causal* role of dysfunctional attentional processing in dyslexia. Similarly, In Kevan & Pammer (2008) dorsal-stream processing deficits were observed in children with a family history of dyslexia *before* they learned to read and Valdois et al. (2019) observed that visual attention performance before children learn to read is related to later reading fluency. Additionally, letter knowledge and measures of dorsal stream functioning could predict early literacy skills (Kevan & Pammer, 2009; see also Peters et al., 2021) In Facoetti et al. (2010) children who had a family history of dyslexia had deficits in both syllabic segmentation and spatial visual attention.

Finally, a potentially related issue is that crowding effects, where visual stimuli are harder to identity when flanked by other elements, have been reported to be larger in children with dyslexia (Bellocchi, 2013; Bertoni et al., 2019; Spinelli et al., 2002). Differences in crowding between dyslexic and normal readers could straightforwardly form a part of a visual explanation for differences in reading ability since stronger crowding can interfere with letter recognition when letters are surrounded by other letters (Gori & Facoetti, 2015). Conversely, reducing crowing by increasing the spacing between letters in words (Perea et al., 2012) or blurring letters (Spinelli et al., 2002; Williams & LeCluyse, 1990; but see Hogben et al., 1996) may help dyslexic readers. The former manipulation could also help by increasing the accuracy of letter position coding (e.g., the difference between “form” and “from”). Crowding has been suggested to be caused to a large part by spatially imprecise focusing of attention (Strasburger, 2005), so problems with crowding and attention in dyslexia could be related.

### 3.3 Hig-Hlevel visual dysfunction hypothesis

A recently developed proposal is inspired by findings that high-level processing of visual information can be atypical in dyslexic readers. High-level visual processing supports visual object discrimination and recognition and reflects activity at later stages of the ventral visual pathway where neuronal activity becomes detached from the retinal image (Cox, 2014). The ventral visual pathway is anatomically and functionally distinct from the previously mentioned dorsal visual pathway (Milner & Goodale, 2006; Ungerleider, 1982; Ungerleider & Haxby, 1994). The high-level visual dysfunction (HLVD) hypothesis is well supported by neuroscientific and behavioral evidence (Sigurdardottir, Ólafsdóttir, et al., 2021).

What does “high-level vision” mean in the context of reading? Neurons in anterior or high-level regions of the ventral visual pathway may selectively respond to complex shapes (Tanaka et al., 1991) and even whole objects (Desimone, 1991), so high-level visual information could roughly correspond to whole words (McClelland & Rumelhart, 1981) or other large units in an orthographic lexicon (Glezer et al., 2009), but also other complex visual features in words. Some high-level visual neurons show tolerance to changes in font or other transformations (e.g., resizing, repositioning) that preserve object structure or identity (Logothetis & Sheinberg, 1996; Pegado, Nakamura, Cohen, & Dehaene, 2011; Zhou, Vilis, & Strother, 2019). While visually presented words rarely share high-level features with other objects, they may still rely on common neural mechanisms. The HLVD hypothesis posits that dyslexic readers may have problems with tasks supported by high-level regions of the ventral visual stream (Sigurdardottir et al., 2015), including impairments with integrating and interpreting features and object parts.

In literates, visually presented words evoke activity in specific areas of the ventral visual pathway, and these regions support visual word recognition (Dehaene & Cohen, 2011). This could reflect connectivity with language areas (Bouhali et al., 2014). Some support for the role of visual properties of words comes from recent work showing that word-selective areas might have a cortical precursor in untrained monkeys – who, of course, have no language (Rajalingham et al., 2019). Neural representations in word-selective ventral stream regions of humans may not be fully abstract as they contain information about common visual formats of words (Wimmer et al., 2016). The authors manipulated the case format of German words (e.g., “haus” instead of the correct capitalization “Haus”), so whether their results are reflect the manipulation of visual features or that the manipulation made words not adhere to German grammar is unclear. Others have however reported that the visual word form area shows some evidence of sensitivity to font (Zhou et al., 2019), again pointing to partial preservation of visual format.

Many findings indicate that people with dyslexia have consistent functional and even structural abnormalities in the ventral visual pathway (see Kronbichler & Kronbichler, 2018; Perrachione et al., 2016; Richlan et al., 2011; van der Mark et al., 2009). Richlan et al. (2011) conducted a meta-analysis of functional imaging studies of reading-related tasks in children and adults with dyslexia and found hypoactivity in high-level ventral stream areas, including the left fusiform gyrus and nearby regions. These abnormalities likely overlap with the so-called visual word form area (VWFA), thought to be involved in reading (Dehaene & Cohen, 2011). The VWFA overlaps partly with other object processing areas in the ventral stream (Grill-Spector & Weiner, 2014; Sigurdardottir et al., 2015), and VWFA activity is not confined to word processing (Price & Devlin, 2003; Starrfelt & Gerlach, 2007). It responds to non-word stimuli such as faces (Dehaene & Cohen, 2011; Nestor et al., 2013) and may become particularly active during fine shape-discrimination of objects (Starrfelt & Gerlach, 2007). Cortical hypoactivity in dyslexic readers appears to extend to other object-selective areas in the ventral stream (Sigurdardottir et al., 2015). Atypical high-level, ventral visual processing might therefore not be restricted to words or word-selective regions.

Dyslexic readers can have difficulties recognizing not only words but also other objects when the task requires fine shape discrimination, including telling apart different exemplars of faces, birds, butterflies, houses, planes, traffic signs, trees, knives, flowers, symbol strings, or abstract figures (Brachacki et al., 1995; Collins et al., 2017; Gabay et al., 2017; Huestegge et al., 2014; Menghini et al., 2010; Sigurdardottir et al., 2015, 2018, 2019). Dyslexic readers might therefore have problems with distinguishing between visually similar objects. Again, consistent with the heterogeneity of dyslexia, this appears to be confined to a subgroup of dyslexic readers. For example, some dyslexic readers appear to have poor face processing abilities while others do not, leading to greater variation in performance for dyslexic than typical readers (e.g., Johnels et al., 2022; Kühn et al., 2021; Sigurdardottir et al., 2018).

Notably, Sigurdardottir et al. (2019) reported that visual recognition problems among dyslexic readers appear to be most prominent in those who have the lowest education levels compared to education-matched typical readers, a result replicated in Jozranjbar et al. (2021). Does this suggest that the dyslexia symptoms of those with lower education levels are more severe? This is certainly possible since severe reading problems may affect children’s academic achievements and their feelings towards the educational system, but readers with more education might also have more reading experience which can affect the visual system (Goswami, 2015). Problems with the visual processing of words could lead to a vicious cycle of non-reading, further affecting both the development of visual processing and reading. High-level visual abilities, unlike some low-level visual abilities (e.g., grating acuity, contrast sensitivity), have a long developmental trajectory (Nishimura et al., 2009), and are very sensitive to experience (Sigurdardottir & Gauthier, 2015), leaving plenty of time and opportunity for interactions with other risk and resilience factors of dyslexia (discussed in section 3.1). Highly educated people with dyslexia are probably more likely to be compensated dyslexic readers, having developed strategies that may leave their visual recognition relatively unaffected as measured behaviorally (but could potentially be detected neurally; unpublished observations, Devillez & Sigurdardottir, 2021). For example, some dyslexic readers may compensate for a visual featural processing deficit by implementing reading strategies that tap into other types of processing such as global or holistic processing (Sigurdardottir et al., 2021, supplementary information).

To further uncover the critical functions involved in high-level deficits in dyslexia, Sigurdardottir, Arnardottir, et al. (2021) measured two types of visual object processing where faces had to be matched based on their features (e.g., the fine-grained shape of their eyes) or their global form (form of the skull, muscles, and fat structure). They reported a dissociation where dyslexic readers performed worse than typical readers on featural matching of faces while there were no group differences for global form face matching. Face and word perception were associated when the processing of visual features of a face was required, while global form processing of faces was not connected to visual word processing at all. This is consistent with the former visual processing mode playing a larger part in visual word recognition (Johnston & McClelland, 1980; Pelli & Tillman, 2007; Wong et al., 2011, 2019) and can help explain some inconsistencies regarding the association or dissociation of face and word processing in the literature. In Jozranjbar et al. (2021) dyslexic readers, unlike typical readers, appeared to rely on a single visual processing mode regardless of whether visual features (e.g., shape of eyes or windows) or their configurations (e.g., distance between eyes or windows) were task-relevant for recognizing faces and houses. Jozranjbar et al. (2021) speculated that reading problems for a subset of dyslexic readers may reflect this reliance on a single visual object recognition process. This process could possibly be holistic. Holistic face processing in dyslexia has been found to be intact (Sigurdardottir et al., 2015; Brady et al., 2021). Tso et al. (2020) demonstrated that dyslexic Chinese high-school students showed stronger holistic processing for Chinese characters than typical readers. Dyslexic readers are also appear more likely to use holistic processing for words in an alphabetical language (Brady et al., 2021). The possibility that visual problems in dyslexia relate to processing mode is intriguing and requires further study.

It is not well understood how visual processing differences relate to other atypicalities of dyslexic readers, such as attentional problems, crowding, and unusual eye movements. For example, Chinese dyslexic readers may have stronger holistic processing of Chinese characters due to difficulties with attending to character parts to form part-based representations (Tso et al., 2020). However, the association between specific problems in featural/part-based processing and reading problems does not seem to be driven by general attentional issues (Sigurdardottir et al., 2021, supplementary information) which some dyslexic readers may still have. Such a connection with attention might hinge on what aspects of attention are measured. Greater crowding in dyslexia (Bellocchi, 2013) could be due to a spatially imprecise focusing of attention (Strasburger, 2005). This could lead dyslexic readers to favor a more holistic processing style requiring a broad attentional window. Blurring letters in words has been shown to have no effect on or even help dyslexic readers, possibly by reducing crowding (Spinelli et al., 2002; Williams & LeCluyse, 1990; but see Hogben et al., 1996). However, blurring may also reduce featural processing while leaving global or holistic processing relatively intact (e.g., Goffaux & Rossion, 2006; Hughes et al., 1990; but see Cheung et al., 2008). These results are therefore consistent with the possibility that dyslexic readers rely on holistic visual processing to compensate for weak featural processing. Finally, unusual eye movements in dyslexia may reflect different processing strategies, including in holistic vs. featural processing. For example, Hautala et al. (2022) tracked eye movements during reading and concluded that holistic orthographic processing of words is likely intact in non-fluent readers.

The relationship between visual processing strategies and reading theories also needs to be better specified. For example, the connectionist multiple-trace memory model for polysyllabic word reading (Ans et al., 1998; Valdois et al., 2004) posits two procedures that support reading: a global procedure where a visual attentional window extends over a whole word, followed by an analytic procedure where a narrower attentional window moves through parts of a word. Hautala et al. (2022) also suggest that readers’ visual word recognition involves a two-stage process: holistic lexical processing of a word followed by a more fine-grained analysis involving grapheme-to-phoneme conversion. This bears some resemblance to how global and fine visual information is encoded by single neurons in high-level regions of the ventral visual pathway (Sugase et al., 1999), with global information being present early, converging later with more detailed information (see also Petras et al., 2019). This stands in contrast with reports of part-based processing of faces preceding their holistic processing (Wang, 2019), which may reflect terminological confusion about global, holistic, featural and part-based processing (see e.g., Richler et al., 2012). Finally, it may be interesting to investigate further how feature-based vs. holistic visual processing relate to differences in reading strategy and teaching methods. We expect that a phonics approach (Castles et al., 2018), which explicitly teaches grapheme-to-phoneme associations, strongly encourages feature-based visual processing as it involves breaking words into individual features or parts.

While not confined to words, high-level visual difficulties may still be relatively specific and involve problems with the development of visual expertise. For example, Gabay et al. (2017) found that readers with dyslexia had problems with face processing, an expert category, but not for cars, a non-expert category, and Sigurdardottir et al. (2018) found problems for faces but not for novel objects. Perceptual expertise has also recently been found to predict reading in dyslexic readers of Chinese, a non-alphabetical language (Wong et al., 2021). This visual expertise deficit however stands in some contrast to the results of Sigurdardottir et al. (2015) who found problems with both faces (considerable expertise) and non-face familiar objects (less expertise) and to Sigurdardottir et al. (2019) who found face recognition problems regardless of experience with faces (own vs. other-race faces). Considerable research does nonetheless indicate that dyslexia involves problems with utilizing previous visual experience (see section below on visual learning and temporal context in perception).

Exactly why dyslexic readers appear to have problems with some objects and not others is still unclear. One possible line of future work would be to further test whether visual object processing problems in dyslexia are overall most apparent for so-called visually ambiguous or crowded categories (object groups where numerous different members are visually similar, not visual crowding effects; Damasio et al., 1982; Gaffan & Heywood, 1993). A high-level ventral stream dysfunction may lead to the use of unusually small feature sets for object identification (Gaffan et al., 1986). This may disproportionately affect objects within a crowded category as they require more precise visual representations for discrimination (Gaffan & Heywood, 1993). Objects of expertise, including visual words, are often such crowded categories. This is consistent with findings where dyslexic readers make more detail-related errors in visual recognition (a potential visual resolution deficit, Huestegge et al., 2014).

In sum, dyslexic readers show consistent abnormalities in the function of high-level regions of the ventral visual stream and have problems with visual tasks that are thought to rely on these regions. Our own work as well that of others shows that developmental dyslexia, a seemingly word-selective deficit, might not be specific to this object category after all. This may, overall, reflect that reading problems of at least a considerable portion of dyslexic readers are influenced by more generalized problems with high-level visual processing. Further work on the potential role of such factors in dyslexia could guide the development of novel screening methods to identify those at risk for developing reading problems, as well as guiding the development of novel training programs for people who struggle with reading. Early detection of the high-level visual deficits that may put children at an increased risk for reading difficulties – before any overt reading problems arise – could provide opportunity for early interventions when brain plasticity is particularly high and cognitive and perceptual training can be expected to have maximum long-term effects. For example, ventral stream fMRI responses to visually presented letters, false fonts, and faces predict future reading impairments of kindergarten children (Centanni et al., 2019; Lieber et al., 2021). Behavioral paradigms that assess prereading children’s high-level visual processing abilities could possibly provide a more cost-effective alternative. Notably, like other risk factors, high-level visual dysfunction may or may not lead to dyslexia, depending on other cumulative risk and resilience factors.

### 3.4 Visual learning and temporal context

Text contains regularities. Certain letter combinations are more likely than others and difficulty with picking up such visual statistical regularities could decrease the fluency of text processing and slow down reading. Sigurdardottir et al. (2017) tested adult Icelandic dyslexic readers and a control group on a visual statistical learning paradigm (e.g., Fiser & Aslin, 2001), finding that dyslexic readers were less likely than their paired controls to pick up which pairs of novel shapes frequently appeared together. Problems with visual statistical learning have also been reported for dyslexic readers of non-alphabetical languages, such as for Chinese dyslexic children where this type of learning predicts how well the children read Chinese words (Tong et al., 2019). Statistical learning is thought to shape neural responses in ventral stream regions (Li & DiCarlo, 2010; Meyer et al., 2014; Turk-Browne et al., 2009) that are important for the visual processing of text and have been found to be hypoactive in dyslexia (Richlan et al., 2011). Sigurdardottir et al. (2017) therefore suggested that problems with visual statistical learning could make neurons in the ventral visual pathway less selective for complex visual features – including letter combinations – which could then affect reading fluency. Statistical learning problems in dyslexic readers have also been reported in the auditory domain across linguistic and nonlinguistic stimuli (Gabay et al., 2015). Dyslexia might therefore reflect deficits in the learning of statistical regularities in the environment more generally. A recent meta-analysis of 49 empirical studies (Lee et al., 2022) concluded that dyslexic readers have domain-general and language-independent statistical learning deficits.

Banai & Ahissar (2018) reported that problems with picking up sound statistics occur in about 50% of people with dyslexia. While they may constitute a subgroup of dyslexic readers, it is unclear whether putative statistical learning problems of dyslexic readers are independent of other suggested problems in dyslexia. For example, they might be mediated by attentional problems (Sigurdardottir et al., 2017). They could also be a manifestation of a more general deficit in procedural learning (Gabay et al., 2015; Nicolson et al., 2010) or reflect lessened effects of recent perceptual information or top-down expectations on current perceptual representations due to dysfunction in rapid neural plasticity (Ahissar, 2007; Beach et al., 2022; Perrachione et al., 2016). Also, some studies have not found any problems at all with visual statistical learning in dyslexic readers (e.g., van Witteloostuijn et al., 2019, 2021; see also discussion in Schmalz et al., 2017) but the meta-analysis by Lee et al. (2022) strongly suggests that the problem is real. It is also possible that people with dyslexia show atypical predictive processing in visual statistical learning paradigms as measured neurally, that does not always manifest in overt behavior (Singh et al., 2018).

Our perceptual systems constantly try to predict the environment using feedback from *prediction errors* to update representations of the external world (Chetverikov & Kristjánsson, 2016; Kristjánsson, 2023; Friston, 2012; Rao & Ballard, 1999), highlighting the importance of temporal context for perception. Problems with such predictive processing could manifest as difficulty in using temporal continuity in visual perception and other modalities. Lieder et al. (2019) tested an auditory serial dependence task (Fischer & Whitney, 2014; Manassi et al., 2019; Rafiei et al., 2021; see Pascucci et al., 2023 for review). They found that individuals with dyslexia rely more on information from the immediate past than controls whose performance reflected longer-term statistics.

The proposal that difficulties in processing of temporal context play a role in dyslexia is very interesting but requires a few extra steps for complete viability. The route to dysfunctional reading is not obvious and some mechanisms must be proposed. One such proposal is the anchoring hypothesis (Ahissar, 2007; see recent discussion in Shulver & Badcock, 2021). The proposal is that people with dyslexia have trouble anchoring to the recent past, which then adversely affects long-term representations (Banai & Ahissar, 2010). This entails that dyslexic individuals fail to benefit from stimulus-specific repetitions (consistent with the findings of Lieder et al., 2019). This can account for phonological, working memory, visual and auditory difficulties, and greater sensitivity to noise, as successful anchoring should aid with deciphering signal from noise. Dyslexia may involve a difficulty linking perception with perceptual memory through the implicit formation of stimulus-specific anchors (Ahissar, 2007). Importantly, temporal processing accounts may be *amodal*, i.e. not visual, auditory or phonological but a general information processing deficit (e.g., Ahissar, 2007; Facoetti et al., 2010; Goswami, 2011; Stein & Talcott, 1999; Hari & Renvall, 2001).

### 3.5. Remaining issues regarding visual dysfunction in dyslexia

How do the various possible problems associated with developmental dyslexia interact, and are they independent of phonological factors? Support for non-phonological accounts of dyslexia comes from studies of dyslexic participants with dyslexia without phonological deficits. Lallier et al. (2013) reported problems with the processing of simultaneous auditory stimuli in dyslexic children irrespective of their phonological symptoms (see also Lallier et al., 2010; Lassus-Sangosse et al., 2008; Peyrin et al., 2012). Such independence from phonological problems has also been used to argue for a specific visual deficit (Bosse, Tainturier & Valdois, 2007; Wolf & Bowers, 1999). Peyrin et al., (2012) reported a double dissociation between phonological and visual attention span disorders both neurally and behaviorally. But visual problems may nevertheless overlap with phonological problems and further understanding of these issues may be reached by assessing the same individuals on numerous tasks that tap different mechanisms.

Finally, it is important to highlight that there is considerable evidence for interventions where training on visual tasks has been found to improve reading performance such as in the use of video games (Franceschini et al. 2012; 2017; Bertoni et al. 2021). In a meta-analysis of game-based training, Ren et al. (2023) reported that the improvements of visuospatial attention enhanced reading fluency in children with dyslexia. They also found moderate evidence that the duration of the benefits could be weeks and recently, Pasqualotto et al. (2022) reported benefits of video game training up to 6 months.

## 4. Multiple causes of dyslexia?

As discussed in section 2, several authors have argued that multifactorial accounts are most likely to explain dyslexia (e.g., Catts & Petscher, 2022; Haft et al., 2016; McGrath et al., 2020; Ozernov-Palchik et al., 2016; Pennington, 2006; Pennington et al., 2012; van Bergen et al., 2014; Vandermosten et al., 2016). These authors argue that the focus on a single disorder is detrimental and that dyslexia should not be considered a discrete condition, but a *label* for difficulty with learning to read. The key point is that the label does not presuppose a particular cause for the problem and dyslexia can even be part of a continuum of normal individual variability in reading ability (Protopapas, 2019). Like we do, Catts & Petscher (2022) criticize the single-cause approach and propose that when assessing risk of dyslexia, multiple factors can be considered, with no single factor necessary or sufficient for the diagnosis. According to McGrath et al. (2020) multiple predictors of dyslexia contribute *probabilistically* to the disorder. Similarly, O’Brien & Yeatman (2021) argue that several distinct, potentially additive, risk factors explain the disorder. Our overview clearly shows that dyslexic readers can have various problems, including in visual tasks and other non-phonological tasks. No single account can explain all the available data which is expected if dyslexia is indeed a multifaceted disorder.

But while evidence has accumulated that visual (as well as other non-phonological) dysfunction plays a role in at least some cases of dyslexia, this general idea has met with surprisingly vocal protest.

Some authors dismiss outright that visual factors play any role in dyslexia (Peterson & Pennington, 2015). In their otherwise highly scholarly overview of dyslexia, Peterson & Pennington pretty much ignore visual factors in dyslexia and mention them only in a dismissive way. They say: “Although it remains possible that some sort of visual processing problems correlate with dyslexia, the scientific consensus for the last several decades has been that dyslexia is a language-based disorder whose primary underlying deficit involves problems in phonological processing (p. 289)”. But appealing to consensus does not make a statement true, and there is no consensus. Furthermore, a majority view can still reflect an oversimplification of a complicated disorder. Peterson & Pennington even argue that visual accounts of dyslexia involve a public health issue, stating: “Unfortunately, the perception that dyslexia primarily reflects a visual problem persists among many in the lay public and continues to form the basis of therapies for the disorder that lack empirical support (p. 289)”. But mentioning pseudoscience and snake-oil salesmen in no way undermines well-supported arguments and empirical data supporting that dyslexic readers have visual problems. Proponents of the phonological account sometimes appear to be playing a game of “whack-a-mole” where they are ready with their mallet whenever proposals of visual problems in dyslexia raise their heads. One need only look so far as to an influential statement from the American Academy of Pediatrics (Lueder et al., 2009) which contains statements such as “[c]urrently there is no adequate scientific evidence to support the view that subtle eye or visual problems cause learning disabilities” (p. 843). As Lack (2010) highlights, this statement is simply untrue.

### 4.1. Revisiting the chicken and egg problem in the development of dyslexia

A far more interesting proposal is that problems with visual perception reflect that dyslexic readers have impoverished experience with the relevant letter and text stimuli due to a lack of training because dyslexic people read less. Visual problems in dyslexia are therefore a consequence rather than a cause and reading is first and foremost a linguistic process (Goswami, 2015). Phonological recoding skills allow children to map sounds, in the form of phonemes, onto visual information in the form of graphemes. Goswami argues that no visual accounts have convincingly demonstrated that apparent visual problems are not caused by reduced reading experience.

While it is undeniable that learning to read induces widespread changes in the visual system, both in the cortex and even in its functional connections to subcortical regions (Dehaene et al., 2015; Skeide et al., 2017), this in no way proves that visual factors do not cause reading problems; if reading development so greatly affects the visual system, this only further emphasizes how heavily reading relies on this system. In fact, it is already well-established that specific reading problems can appear after damage to the visual system (Adler, 1944, 1950; Sparr et al., 1991). And just like people with developmental dyslexia, patients with acquired reading problems can have problems with other visual tasks, such as judging whether line drawings contain real or nonsensical objects (Starrfelt et al., 2010). At the other end of the spectrum, there are people with superior reading abilities – perhaps akin to the difference between people with developmental prosopagnosia (severe face recognition problems) and super-recognizers (unusually efficient face recognition; see e.g. Russell et al., 2009; but see Hendel et al., 2019). In the end it would be surprising if individual differences in the functioning of such an important system would not lead to reading problems as in developmental dyslexia.

An additional important issue is that *critical periods* exist for various basic visual mechanisms (Harwerth et al., 1986), including many functions that are presumably affected in dyslexia according to visual accounts. Notably, these critical periods occur very early, typically before children start to read. This is highly problematic for any account that postulates that visual problems are the result of impaired reading abilities, such as from the lack of reading experience. For example, critical periods for the development of binocularity peak between 1 and 3 years of age, and early corrective surgery for esotropia seems necessary for the development of cortical binocularity (Banks et al., 1975; Simonsz & Kolling, 2011), which presumably is a prerequisite for successful binocular fusion and stereopsis. Note also that functions such as flicker sensitivity and contrast sensitivity reach adult levels very early, becoming roughly adult-like before children have much experience with reading (Norcia et al., 1990; Regal, 1981). Although there is considerable plasticity in the brain (Kristjánsson et al., 2016; Sigurdardottir & Gauthier, 2015), potential mechanisms for learning effects from reading upon specific brain function must be clearly specified, especially in cases of low-level visual mechanisms.

### 4.2. Multifactorial accounts of dyslexia and the developmental perspective

While we believe that visual factors could play a causal role in some cases of dyslexia, it would be naïve to assume that all relations are one-way. The relationship between reading and visual function is likely to involve a two-way street – dysfunctional visual processing leads to problems with reading that can then be exacerbated by lessened exposure to reading materials, in a vicious cycle. A better way of thinking about dyslexia and how it develops is in terms of an interlocking network that is dysfunctional at one or more stages and throughout development these mechanisms interact. We agree with Goswami’s (2015) call for longitudinal studies that may be needed to satisfactorily address causality in dyslexia. Well-designed longitudinal studies are often the only way of clearly assessing causality regarding accounts of developmental disorders and how training programs can alleviate problems or improve function (Kristjánsson, 2013). A few such studies do exist. For example, Centanni et al. (2019) found that left fusiform regions of the ventral visual pathway were hypoactive in pre-reading children who later became impaired readers; this was not only true for text but was also found when the children viewed letter-like stimuli and images of faces. Liebig et al. (2021) followed 54 children from before they began to read until 2 years after formal reading training started. They found that stronger neural response to faces in the ventral stream was longitudinally associated with better reading performance. Also, Raschle et al. (2012) found that some posterior brain regions were hypoactive in prereading children with a family history of dyslexia. While the authors interpret these results in relation to phonological processing, we note that at least some of the identified regions might be primarily visual (e.g., compare hypoactive regions in Raschle et al., 2012; their figure 1C, and locations of the object-selective lateral occipital complex and the adjoining motion-selective area V5/MT+, see e.g. figure 1 from Malach et al., 1995). Lower activity in these regions was found *prior* to formal reading instruction and could therefore play a *causal* role in reading problems.

How should we consider the development of dyslexia in light of the evidence for visual dysfunction in dyslexia and the multifactorial context that we propose? We suggest that the most straightforward way is to take an interactive approach to how dyslexia develops. This approach should consider dyslexia from a multifactorial viewpoint. Certain deficiencies, such as in phonological awareness or in visual processing, can set a process in motion that may lead to dyslexia, and risks of dyslexia can be strengthened or weakened depending on other resources. As many have highlighted, interactions are almost certain to occur. Children could, for example, be resilient to risk factors of dyslexia from visual problems if they have good phonological abilities and vice versa (Catts & Pletscher, 2022). There is indeed evidence that risk factors for dyslexia can be additive (Catts et al., 2017; O’Brien & Yeatman, 2021) or contribute probabilistically (McGrath et al., 2020). Importantly, visual problems that may lead to dyslexia can manifest very early during development and critical periods for various visual functions can occur before children start learning to read. Speedy identification of such problems should therefore be of benefit to reading development.

Another factor that we can only briefly discuss here is that since dyslexia is highly heritable, this could allow identification of genetic propensity for dyslexia (Peterson & Pennington, 2015). Erbeli, Rice & Paracchini (2022) recently concluded, firstly, that dyslexia is highly polygenic. Secondly, genetic risk factors seem to overlap with other neurodevelopmental disorders such as attentional disorders, language disorders, and dyscalculia. For example, a specific gene variant has been related to both dyslexia and dyscalculia (Úlfarsson et al., 2017). Doust et al. (2022) revealed a quite heterogeneous genetic profile associated with dyslexia. Overall, the evidence from genetics is highly consistent with multifactorial accounts.

## 5. Conclusions

Our argument consists of 5 main points:

- There can be many causes of a similar behavioral pattern, and there is no core deficit that accounts for all cases of dyslexia. Dyslexia may involve problems with phonology, with visual processing or more general processing deficits that supersede modalities such as with temporal integration and context; and these deficits could show considerable overlap. Dyslexia is probably caused by different combinations of these factors for different individuals.
- Many people who have dyslexia also have symptoms other than problems with reading, and visual problems are very prominent among them. This raises the possibility that dyslexic readers could be subdivided into groups with different causes of dyslexia, some of which could be primarily visual. An important challenge for understanding the development of dyslexia and for its treatment is to uncover which disorders cause which symptoms and which treatments affect which subtypes of dyslexia.
- Consistent with the complexity of the human visual system, putative visual factors in dyslexia come in many flavors. Arguments against one do not necessarily have any weight in the dismissal of the role of other visual factors. Vision is not just one thing.
- Proposals that visual problems in dyslexia reflect differences in reading experience are not fundamentally in opposition to the possibility that visual cognition plays a causal role in the disorder. Both can simultaneously occur because development is a two-way street.
- We argue for a multifactorial view of dyslexia and longitudinal studies are needed where the potential contribution of problems with visual mechanisms to dyslexia can be dissociated from the influences of other factors.

No single account of dyslexia has turned out to be quite satisfactory which probably explains the dissent in the field. And a large set of research findings is available that often point in different directions. A heterogeneous set of symptoms literally *argues for* a multifaceted conception of the etiology of dyslexia and can explain mixed findings in the literature. The proposal that visual problems may explain dyslexia has met with fierce opposition for decades, opposition that we believe is misguided and reflects a restrictive single-cause approach to understanding dyslexia. We emphasize that we do not wish to argue against phonological accounts of dyslexia *per se*. We however believe that multifactorial accounts involve the most parsimonious explanation for the varied symptoms.

Reading is one of the most complex forms of information processing in humans (e.g., Lesgold & Perfetti, 1981). This also means that the process can go wrong in many ways, bringing us back to the analogy of the passenger who insists that only a single reason can explain the malfunction of the car stopped on the highway. 30 years ago, Slaghuis et al. (1993) wrote: “…despite evidence for the involvement of perceptual factors in the etiology of dyslexia the prevailing view is that the disorder is almost entirely due to language related difficulty“. It is interesting to compare this to what Raghuram et al., say in 2018 (Raghuram et al., 2018): “…little is known about the integrity of visual function in [developmental dyslexia]“. This sounds as if little progress has been made in 25 years and the story of research into dyslexia has a strong ring of déjà vu about it.

We believe that denial of any role for visual processing deficits in the etiology of dyslexia explains this illusory sense of a lack of progress. There is strong evidence that problems with visual perception contribute to dyslexia symptoms and the evidence keeps stacking up, such as for the high-level visual dysfunction hypothesis (section 3.3). Advocates of strong phonological views that dismiss any role of visual factors are in fact discounting a large amount of relevant evidence that will aid the understanding dyslexia, ultimately helping those who have difficulties with reading. We hope to have convinced the reader that the role of visual factors in dyslexia cannot be easily dismissed.

### 5.1. What next?

In the end, “developmental dyslexia” may become an obsolete term, some sort of phlogiston, as it is likely not just one thing but many different things with various causes. Catts and Petscher (2022) emphasize that dyslexia can be seen as a label or a synonym for an unexpected reading problem, a problem that can be due to many factors but not the name of a single core neural deficit. This is consistent with evidence that reading ability can be considered as a continuum from poor to strong reading skills (Protopapas, 2019). Just as there are no clear cutoffs for being “short” or “tall”, there may also not be any cutoff for being “dyslexic” – both could be the lower end of a continuum of attributes that are simultaneously influenced by many factors. We should aim to identify risks that may be apparent before reading instruction and try to address those. Visual factors almost certainly play a role, but importantly different individuals are likely to have different risk and resilience factors. Some but not all dyslexic readers may have visual problems, and not all dyslexic readers may have the same visual problems. This poses challenges. Who are the dyslexic readers showing visual deficits? What visual properties do they have difficulties with? Are their visual problems a cause of their reading disorder, a consequence, or both? Studies where different factors are assessed and contrasted with one another seem crucial for moving forward. Recent examples of such studies have been consistent with multifactorial accounts (Ramus et al., 2003; White et al., 2006; Saskida et al., 2016).

Additionally, large-sample longitudinal studies with diverse test batteries are needed to draw firm conclusions regarding subtypes of dyslexia, what their causes are, which contribute independently and which ones overlap, and what measures – neural and behavioral – are best for identifying causes at an individual level. This would involve large effort, even adversarial collaborations across countries as some effects may be language-dependent (Norton et al., 2015; Ziegler et al., 2010). Such coordinated effort seems nevertheless small considering the effort that has been put into research on dyslexia over several decades. We believe that visual function will form a large part of a future account of reading problems where diverse interacting causes are acknowledged.
